# The Emergence of Modern Languages: Has Human Self-Domestication Optimized Language Transmission?

**DOI:** 10.3389/fpsyg.2018.00551

**Published:** 2018-04-17

**Authors:** Antonio Benítez-Burraco, Vera Kempe

**Affiliations:** ^1^Department of Spanish, Linguistics, and Theory of Literature, Faculty of Philology, University of Seville, Seville, Spain; ^2^Division of Psychology, School of Social and Health Sciences, Abertay University, Dundee, United Kingdom

**Keywords:** language evolution, language acquisition, self-domestication, Linguistic niche hypothesis, child-directed speech

## Introduction

Our uniquely human ability to learn and use languages (aka *language-readiness*) has been hypothesized to result from species-specific changes in brain development and wiring that habilitated a new neural workspace supporting cross-modular thinking, among other abilities (Boeckx and Benítez-Burraco, 2014; see Arbib, 2012, 2017 for a similar view). Strikingly, behavioral modernity did not emerge on a par with cognitive modernity. On the contrary, it is only well after our split from Neanderthals and Denisovans that modern behavior becomes evident around the world (see Mellars et al., 2007; but also Hoffmann et al., 2018; for tentative evidence of behavioral modernity in Neanderthals). This emergence of modern behavior has been linked to the rise of modern languages, i.e., exhibiting features such as elaborate syntax including extensive use of recursion. The potential of these languages to convey sophisticated meanings and know-how in ways that allows sharing of knowledge with others is assumed to have arisen in a reciprocal relationship with complex cultural practices (Sinha, 2015a,b; Tattersall, 2017). Thus, even if not its main trigger, complex language is at the very least a by-product and facilitator of modern behavior.

**Figure 1:**
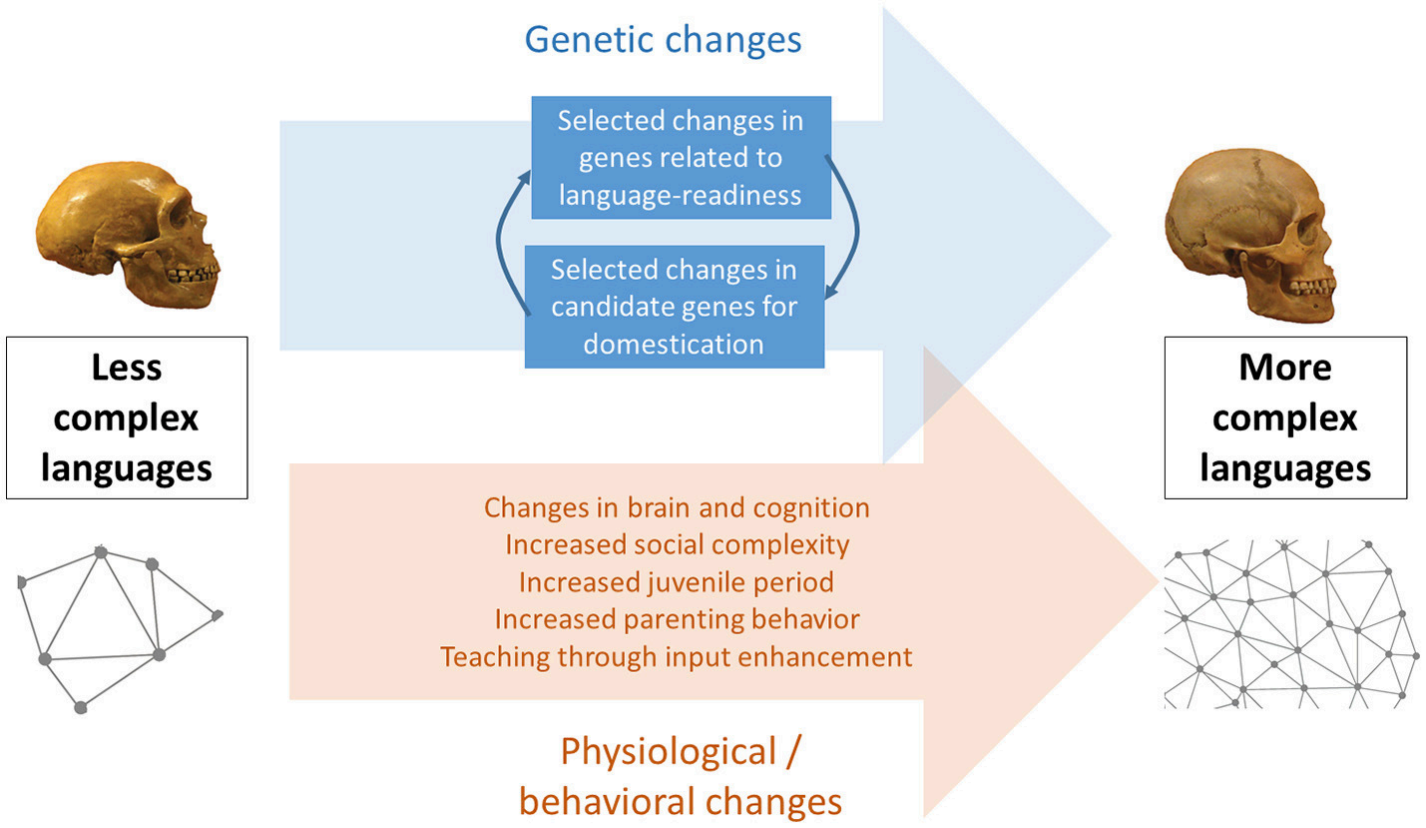
A graphical summary of the hypothesis of human self-domestication as a key factor in the optimization of language transmission and ultimately, in the emergence of modern languages. The skulls from Neanderthals **(left)** and AMHs **(right)** are from Boeckx and Benítez-Burraco (2014).

Because the human brain and human cognition have remained substantially unmodified since our origins, behavioral modernity and modern languages are assumed to be the product of cultural evolution via niche construction (Sinha, 2009, 2015b; Fogarty and Creanza, 2017). This may include feedback effects of culture on our cognitive architecture in the form of the creation of “cognitive gadgets” (Clarke and Heyes, 2017) through small modifications in learning and data-acquisition mechanisms like attentional focus or memory resources (Lotem et al., 2017), but without involving significant neuro-anatomical changes (Figure 1). However, this explanation may be insufficient: Recent research suggests that aspects of the human distinctive globular skull and brain might have evolved gradually within our species in response to accompanying genetic changes, reaching present-day human variation between about 100 and 35 thousand years ago (kya), in parallel with the emergence of behavioral modernity (Neubauer et al., 2018). Thus, it may not be entirely appropriate to equate neuro-anatomical modernity with cognitive modernity; instead, the language-ready brain can be conceived of as a brain with the potential for cognitive modernity. Here we argue that these neuro-anatomical and concomitant behavioral changes are largely manifestations of human self-domestication, which constitutes a possible pathway toward cognitive modernity and sophisticated linguistic abilities. We focus specifically on parenting and teaching behaviors as foundations of cultural transmission processes that may have facilitated the exploitation of our cognitive potential and, ultimately, the emergence of modern languages.

## Characteristics of modern languages

There is discussion about which aspects of linguistic structure are part of the biological endowment and which ones are products of cultural transmission. On the one hand, complex phonologies, opaque morphologies (with more irregularities and morpho-phonological constraints), limited semantic transparency of lexical items and formulaic idioms, reduced compositional structure, and less sophisticated syntactic devices are found among isolated human groups living in small, close-knit communities with higher proportions of native speakers who share considerable amounts of knowledge (so-called *esoteric* communities). According to the Linguistic Niche Hypothesis (Lupyan and Dale, 2010), these linguistic features are readily learnable by immature learners who often rely, as children do, on an item-based learning process that takes under-segmented multi-word utterances as its input (Tomasello, 2000; Arnon and Christiansen, 2017). The obligatory morphological marking by multiple features found in such opaque morphologies results in overspecification (McWhorter, 2007; Trudgill, 2011; Caballero and Kapatsinski, 2015), which might help child learners to correctly identify and predict core components of utterances to establish who did what to whom. Finally, the presumed greater extent of common ground may reduce the need for rapid context-dependent online disambiguation, which is problematic for children (Trueswell et al., 1999; Snedeker and Trueswell, 2004).

On the other hand, subsequent increases in social complexity involving larger population size, greater rates of inter-group economic transactions and cross-cultural exchange, increased polity size, greater division of labor, increased hierarchical social organization and, more recently, the invention of writing systems (Turchin et al., 2017), may have shifted the emphasis from esoteric language use as a joint action tool toward language as a means of decontextualized information transmission between unfamiliar individuals in so-called *exoteric* communities. This would have required an expansion of vocabularies, and an increase in syntactic complexity (including greater reliance on recursion, see Everett, 2005). The greater cognitive cost to language processing and learning incurred by this expansion might partially be alleviated by simpler sound combinations, more regular morphologies, greater compositionality, and enhanced semantic transparency (see Bolender, 2007; Wray and Grace, 2007; Lupyan and Dale, 2010; Trudgill, 2011; Nettle, 2012 for discussion). Thus, we do not view exoteric morphologies as merely simpler than esoteric morphologies, as suggested by others (Szmrecsanyi and Kortmann, 2009; Lupyan and Dale, 2010; Bentz and Winter, 2013; Reali et al., 2018). Instead, we suggest that the main difference between exoteric and esoteric communication systems lies in their context-dependency. Specifically, in exoteric communities, the need for decontextualized language use may have driven morphological structure toward greater informativeness based on degree of transparency and regularity to support greater communicative efficiency, e.g., via morphological devices that mark long-distance agreement patterns or that allow for immediate thematic role assignment, to handle the increased lexical and syntactic complexity needed for more sophisticated information transmission. At the same time, the drive toward reduced cognitive effort may act to put a cap on morphological richness, i.e., on the number of obligatorily marked grammatical features, compressing morphological paradigms so as to result in some degree of inflectional neutralization and syncretism (van Trijp, 2013). The extant morpho-syntactic variation found in modern exoteric languages presumably reflects different solutions to the trade-off between communicative efficiency of morphological systems given lexical and syntactic complexity and the cognitive effort required for processing these systems (Piantadosi et al., 2012; Kemp et al., 2018). The results of such a trade-off may be captured through information-theoretical descriptions of morphological cues (e.g., Bates and MacWhinney, 1989). This view makes the predictions—to be tested in future research—that across languages, social complexity should be positively linked to syntactic/lexical complexity, and syntactic/lexical complexity should be linked to greater informativeness of morphological cues,. Because lexical and syntactic complexity is taxing on cognitive resources this view also implies that learning decontextualized exoteric languages requires considerable working memory capacity, executive control and declarative knowledge–resources that are more developed in cognitively mature adult learners (Braine et al., 1990; Brooks et al., 1993, 2006). As a result, the structural features associated with decontextualized language use may introduce the need for input enhancement and scaffolding of language acquisition for cognitively immature children.

According to Wray and Grace (2007), esoteric languages are the default linguistic systems of humans groups. Ancient DNA studies have recently shown that the social organization of anatomically-modern humans (AMHs) living around 34 kya resembled those formed by present-day hunter-gatherers (Sikora et al., 2017). The languages spoken at that time are far beyond the limits of the best linguistic reconstructions (Nichols, 1997). However, the parallels in social structure suggest that prehistoric communities of AMHs might have spoken languages containing most of the features of esoteric languages. Importantly, the social systems of contemporary hunter-gatherers are quite diverse, involving different degrees of complexity and interconnection, ranging from sparsely to highly connected systems, with complex hunter-gatherer societies exhibiting a high degree of sedentism, territoriality, elaborated technologies, social stratification, and long-distance exchange (Kelly, 1995; see also Solich and Bradtmöller, 2017 for an evolutionary model of hunter-gatherer societies with connectedness as a key concept). Similarly, prehistoric hunter-gatherer societies were thought not to be homogeneous either: From the late Aurignacian (circa 35 kya) to the late Magdalenian (circa 15 kya) we observe a trend toward increasing mobility, more complex networks, and more complex social bonds. Accordingly, the Aurignacian peoples from Sikora et al.'s (2017) study were organized in small groups with limited kinship and with relatively wide social networks, whereas peoples from the Magdalenian period already maintained complex social systems and extensive trade networks, including periodical meetings of regionally dispersed groups (Conkey, 1980; Schwendler, 2012). For that reason, the opposition between esoteric and exoteric communities (and languages), both at present and in prehistoric times, should be construed not as categorical, but as continuous, and transitions from esoteric to exoteric niches will have occurred under suitable circumstances, particularly in prehistoric times.

That said, the patterns of social complexity of past and present AMH hunter-gatherer groups are in notable contrast with, on the one hand, what can be inferred for other hominin's species and for our own species before 100 kya, and, on the other hand, what has been observed in farming populations and technologically advanced societies. This is supported by archaeological and genetic evidence, which suggests that Neanderthals were organized in small communities of few familial units with low genetic diversity, almost no signals of aggregations, very limited intergroup contacts, and patrilocal mating behavior (Wynn and Coolidge, 2012; ch. 4 and references therein; Lalueza-Fox et al., 2010), which can be construed as strongly-knit esoteric networks. Similarly, as noted, no widespread signals of behavioral modernity have been observed before 100 kya in our species either. In contrast, farming populations emerging around 10 kya (Borrell et al., 2015), exhibit a much higher degree of technological sophistication, social stratification, outgroup contacts, long-distance exchange, and network complexity (all of them features of exotericity), and particularly, of cultural niche construction. It has been suggested that niche construction is a particularly important factor in exoteric societies (Odling-Smee and Laland, 2009; Sinha, 2009, 2015a,b; Pinker, 2010) but, as noted by Solich and Bradtmöller (2017 p. 115), plays a smaller role in esoteric hunter-gatherer societies.

Many different factors driving the increase in socio-economic complexity of prehistoric hunter-gatherer societies, and ultimately, the transition from esoteric to exoteric societies, have been proposed, ranging from environmental factors and demographical changes to new population dynamics (Vaesen, 2012; Borrell et al., 2015; Solich and Bradtmöller, 2017). Below, we focus on self-domestication as a driver not just for increased social complexity including the transmission mechanisms required to maintain the associated linguistic complexity.

## Self-domestication provides pathways to linguistic modernity

As noted above, changes in the human social environment seem to account for how and when exoteric languages emerged. Some authors have suggested that human self-domestication contributed to such changes. The idea that human beings are domesticated primates can be traced back to Darwin (1871). Because no external domesticating agent can be found, this is commonly referred to as the *self-domestication hypothesis*. Among other things, self-domestication might have favored the creation of a cultural niche that permitted the exploitation of the full cognitive potential of our language-ready brain, allowing us to accommodate linguistic structures that require considerable cognitive capacity, thereby increasing language complexity via a cultural process (Thomas, 2014; Benítez-Burraco et al., 2016a).

Domestication gives rise to a constellation of common features in most domestic strains of mammals, as well as in birds, including changes in pigmentation, shorter reproductive cycles, neoteny, changes in the craniofacial area, smaller brains, increased tameness and sociability, and even changes in cognitive abilities (Wilkins et al., 2014; Sánchez-Villagra et al., 2016; Benítez-Burraco, 2017; Okanoya, 2017; Agnvall et al., in press). Interestingly, parallels between domestication and the sophistication of the communicative repertoire have been noted too. Thus, domesticated varieties of songbirds develop more complex songs compared to their wild conspecifics because of the relaxing of selection pressures associated with domestication (Takahasi and Okanoya, 2010; Kagawa et al., 2014; Okanoya, 2017).

Morphological signatures of domestication are prominent in AMHs compared to extinct hominins, including changes in the face, the skull, dentition, neoteny, and reduced aggressiveness (Márquez et al., 2014; Thomas, 2014; Fukase et al., 2015; Stringer, 2016). The expression of features of domestication seems to have intensified since the time when first evidence of modern behavior is conspicuous, between 100 and 50 kya. As noted, the AMH skulls and brains have been globularizing over time, but have been also reducing in size from the last 40 thousand years (Bednarik, 2014). Interestingly, candidate genes for domestication in mammals appear to be enriched in regions under positive selection in AMHs compared to extinct hominins (Theofanopoulou et al., 2017).

Because of the attested link between domestication and the sophistication of communication signals in animals, we should expect some effect of self-domestication on human language abilities. Interestingly, genes that are hypothesized to have played a role in the evolution of our language-readiness are found among, or are functionally connected to, candidates for domestication in mammals (Benítez-Burraco et al., 2016a). Intra-species variability in humans also supports a link between features of domestication and features of language. Thus, conditions like schizophrenia or autism spectrum disorder exhibit both an abnormal presentation of traits ascribed to domestication (Benítez-Burraco et al., 2016b, 2017) and reduced language complexity, in particular, lower syntactic complexity (Fraser et al., 1986; Thomas et al., 1987; Tager-Flusberg et al., 1990).

While self-domestication *per se* may have brought about subtle changes in brain structure and function that contributed to language complexity directly (see Benítez-Burraco, 2017, for discussion), we suggest that it is the less aggressive behavior associated with domestication which served as the main prerequisite for the increase in language complexity. The reason is that a greater sociability enhances the intergroup contacts that ultimately require more complex, cognitively demanding linguistic systems to serve the resulting expanded social networks. Yet only in conjunction with another key consequence of domestication, namely, the increase in neotenic features that sustain extended juvenility (see Hare, 2017, for details), can these systems be learned. These two consequences of self-domestication—prolonged childhood and complex social networks—are thought to give rise to an emerging developmental niche (Sinha, 2015a) through creation of a culture of apprenticeship that ensures transmission of cultural and cognitive capital (Sterelny, 2011). With respect to language, this developmental niche affords rich linguistic interactions that ensure mastery of increasingly more complex decontextualized languages through forms of teaching that build on human mimetic abilities like demonstration (Gärdenfors, 2017) and input enhancement (Shafto and Goodman, 2008) by parents and other caregivers. Thus, our main point is that the impact of self-domestication on language complexity was exerted through a developmental niche that facilitated learning through teaching.

The benefits from spontaneously occurring linguistic demonstration and input enhancement by caregivers for child language acquisition are well documented (for reviews see Soderstrom, 2007; Saint-Georges et al., 2013; Golinkoff et al., 2015). Child-directed speech aids the acquisition of phonology (Liu et al., 2003; but see Martin et al., 2015), word segmentation (Kempe et al., 2005; Thiessen et al., 2005), morphology (Kempe and Brooks, 2005) and especially vocabulary (Ma et al., 2011; Cartmill et al., 2013; Newman et al., 2016; Ota and Skarabela, 2016; Foursha-Stevenson et al., 2017). While the link between parental input and child language development may in part reflect heritability of verbal intelligence (Dale et al., 2015), there is evidence for a causal component in the relationship between parental input enhancement and learning outcomes (Huttenlocher et al., 2007). A number of mechanisms mediate this benefit: aside from boosting children's general language processing skills (Weisleder and Fernald, 2013), child-directed speech can provide an enriched database from which to extract relevant information, especially with respect to lexical development, which, in turn, supports acquisition of syntactic complexity (Marchman and Bates, 1994).

Although these caregiver adjustments in child-directed speech may have their origins in universal hominin caregiving behaviors (Falk, 2004; Broesch and Bryant, 2015; Kalashnikova et al., 2017), the intensity of such behaviors appears to vary along the esoteric-exoteric continuum. Indeed, a broad-range of beneficial input adjustments have been widely documented for exoteric languages spoken in present-day industrialized societies (Fernald et al., 1989; Kuhl et al., 1997; Piazza et al., 2017). In contrast, some evidence suggests that language input to children is limited and speech adjustment by caregivers is reduced in present-day hunter-gatherer societies likely to engage in more esoteric communication (Bavin, 1992; Lieven, 1994; Ochs and Schieffelin, 1995; Cristia et al., 2017). The idea of reduced input enhancement in esoteric societies is indirectly supported by evidence for cross-cultural differences in other aspects of parenting. Thus, depending on the culture, contingent parental reactions reinforce a range of culturally diverse behavioral repertoires of infants (Bornstein et al., 2017), with parental encouragement of infant physical activities and motor skills being less (Karasik et al., 2010, 2015), and didactic activities encouraging cognitive and linguistic skills being more prominent in contemporary Western (i.e., exoteric) societies. Due to methodological difficulties associated with obtaining data on parenting behaviors for large numbers of different societies (Kline et al., 2018) it is at present not possible to reliably link differences in parenting strategies to differences in the social complexity associated with exoteric communication. However, we speculate that input-enhancing child-directed speech is more frequent in exoteric communities where linguistic sophistication can boost social prestige and economic success of individuals. We suggest, thus, that when children need to acquire complex exoteric native languages, they benefit from the extended socialization period, and the enriched interaction patterns enabled by self-domestication, including demonstration, input enhancement, scaffolding of communication and explicit teaching by adults, which might well be indispensable for mastering lexically and syntactically complex exoteric linguistic systems.

There is also comparative evidence that domestication enhances caring behaviors directly, thereby supplying another pre-requisite for teaching behaviors that we assume to support the acquisition of exoteric languages. For example, de-domestication of social species, like free-ranging scavenger dogs, usually results in selfish behaviors by mothers against litters (Paul et al., 2015). Likewise, social isolation of domesticated laboratory rodents impacts on mother-offspring relationships and playing behavior of the pups, ultimately affecting behavioral and cognitive performance in the adult state (Arakawa, 2017). From a developmental perspective, poorer parenting outcomes have been observed in people with schizophrenia (Abel et al., 2005), which, as noted, is a condition that entails reduced language complexity and abnormal self-domestication features. Finally, from an evolutionary perspective, Neanderthals, who arguably had less complex languages than AMHs (Johansson, 2015), have been hypothesized to exhibit briefer childhoods (Smith et al., 2010), more in-group-focused and strongly-knit social networks resulting in socialization patterns focused on internal rather than external bonds (Spikins et al., 2014), and a learning mode based mainly on imitation (Hawcroft and Dennell, 2000). These features support the conjecture that Neanderthal parenting behaviors differed notably from those found in AMHs in terms of amount of demonstration and input enhancement, thus presumably restricting the amount of linguistic complexity that they could acquire.

## Conclusions

To summarize, we argue that human self-domestication created core opportunities for the cultural evolution of cognitive enhancements that hitherto were not fully exploited in human societies. Not only may self-domestication have directly contributed to the linguistic differences between AMHs and extinct hominins in parallel with (or even contributing to) globularization, but around 100–50 kya enhanced domestic features in our species also facilitated the emergence of the social and technological complexity of exoteric societies. At the same time, the extended juvenile period and enhanced parenting brought about by self-domestication supported teaching behaviors that facilitate learning of the complex linguistic systems that underpin behavioral modernity.

## Author contributions

AB-B and VK conceived and wrote the paper.

### Conflict of interest statement

The authors declare that the research was conducted in the absence of any commercial or financial relationships that could be construed as a potential conflict of interest.
